# Fine-scale genomic tracking of Ross River virus using nanopore sequencing

**DOI:** 10.1186/s13071-023-05734-z

**Published:** 2023-06-06

**Authors:** Ellen M. de Vries, Noel O. I. Cogan, Aneta J. Gubala, Brendan C. Rodoni, Stacey E. Lynch

**Affiliations:** 1 Agriculture Victoria, AgriBio, Centre for AgriBioscience, Bundoora, VIC 3083 Australia; 2 School of Applied Systems Biology, La Trobe University, Bundoora, VIC 3083 Australia; 3 Sensors and Effectors Division, Defence Science & Technology Group, Fishermans Bend, VIC 3207 Australia

**Keywords:** Tiled amplicon sequencing, Ross River virus, Nanopore, SNP analysis, Bioinformatics

## Abstract

**Background:**

Ross River virus (RRV) is Australia’s most common and widespread mosquito-transmitted arbovirus and is of significant public health concern. With increasing anthropogenic impacts on wildlife and mosquito populations, it is important that we understand how RRV circulates in its endemic hotspots to determine where public health efforts should be directed. Current surveillance methods are effective in locating the virus but do not provide data on the circulation of the virus and its strains within the environment. This study examined the ability to identify single nucleotide polymorphisms (SNPs) within the variable E2/E3 region by generating full-length haplotypes from a range of mosquito trap-derived samples.

**Methods:**

A novel tiled primer amplification workflow for amplifying RRV was developed with analysis using Oxford Nanopore Technology’s MinION and a custom ARTIC/InterARTIC bioinformatic protocol. By creating a range of amplicons across the whole genome, fine-scale SNP analysis was enabled by specifically targeting the variable region that was amplified as a single fragment and established haplotypes that informed spatial-temporal variation of RRV in the study site in Victoria.

**Results:**

A bioinformatic and laboratory pipeline was successfully designed and implemented on mosquito whole trap homogenates. Resulting data showed that genotyping could be conducted in real time and that whole trap consensus of the viruses (with major SNPs) could be determined in a timely manner. Minor variants were successfully detected from the variable E2/E3 region of RRV, which allowed haplotype determination within complex mosquito homogenate samples.

**Conclusions:**

The novel bioinformatic and wet laboratory methods developed here will enable fast detection and characterisation of RRV isolates. The concepts presented in this body of work are transferable to other viruses that exist as quasispecies in samples. The ability to detect minor SNPs, and thus haplotype strains, is critically important for understanding the epidemiology of viruses their natural environment.

**Graphical Abstract:**

**Figure:**
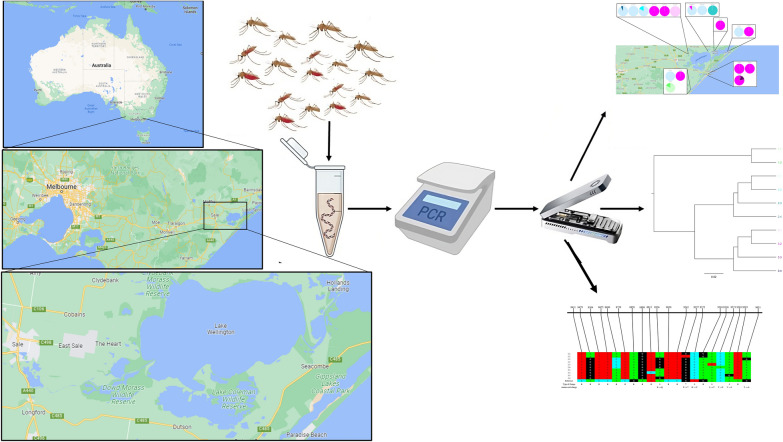

**Supplementary Information:**

The online version contains supplementary material available at 10.1186/s13071-023-05734-z.

## Background

*Ross River virus* (RRV) (genus *Alphavirus,* family *Togaviridae*) is a mosquito-borne arbovirus that is endemic to Australia and also detected in Papua New Guinea and the South Pacific Islands [1]. RRV can cause polyarthritis in humans, a debilitating form of arthritis that has the potential to cause long-lasting health issues [1]. RRV also presents with symptoms such as fever, rash, and fatigue in humans [1]. Clinical signs in horses have also been reported [2] and they have been implicated as amplifiers of the virus [3]. Key mosquito vectors of the virus in Australia are *Aedes camptorhynchus*, *Ae. vigilax*, *Culex annulirostris* [4], and *Ae. notoscriptus* [5]. Macropods have been attributed as the main reservoir host but other placental mammals and birds could also act as reservoirs [6]. The transfer of the virus is spread through the bite of a female mosquito, and occasionally an infected female mosquito can vertically transmit the virus to her eggs, referred to as transovarial transmission [7–9]. Human population expansion into mosquito habitats is increasing human-mosquito interactions [10–12] and increasing the risk for humans to be affected by arboviruses (including RRV) [13]. This worldwide trend is predicted to increase [14, 15], and monitoring of mosquito-borne viruses is therefore imperative to support public health.

RRV was first detected in 1958, near Townsville, in far north Queensland, Australia, with the oldest isolate (T48) being classified as G1 [16]. There are four main genotypes of RRV, with G3 and G4 being direct decedents from G1, and G2 its own separate off group [17, 18]. Each of these genotypes either currently occupy or have historically occupied specific regions of Australia. G1 and G2, although apparently no longer circulating, were commonly found in Queensland and Western Australia in the years pre-2000 [19]. G3, another historical genotype, was predominately located in the Cook Islands during the late 1970s and early 1980s [19]. Some G3 sequences were seen in various Australian states into the early 2000s, with the most recent detection a 2014 isolate from Tasmania. These three genotypes have been mostly replaced by the most common currently circulating genotype, G4. The G4 strain of RRV is divided into sublineages, determined by nucleotide similarity analyses [19]. The G4 sublineages, named G4A, G4B, G4C and G4D, have been reported in Western Australia, Victoria, Queensland, New South Wales, Papua New Guinea and the Northern Territory, with the most recent isolates (2018) assigned to the G4B and G4A sublineages [19].

RNA viruses, such as RRV, mutate rapidly because of the absence of 3' exonuclease proofreading mechanism of their RNA-dependent RNA polymerase [20–22]. The exonuclease activity of the polymerase enzyme plays an important role in nucleic acid replication whereby on recognition of an incorrectly incorporated base, the polymerase’s direction is reversed, and the incorrect base is removed. RNA viruses lack this activity and therefore any incorrectly incorporated bases will remain in the nucleic acid sequence, resulting in a higher rate of mutations in RNA viruses compared to other organisms, although some mutations will result in deleterious mutations [23, 24]. Highly variable regions, including those nucleotide sequences that encode envelope glycoproteins and interact with host cell receptors, are often used for characterisation of viral variants including RRV [19, 25, 26]. In RRV, a commonly used region to inform molecular epidemiological studies is the E2/E3 regions encoding for surface receptor glycoproteins [19, 27, 28]. Higher levels of mutation are often seen in viral glycoproteins, with amino-acid changes in these regions linked to increases in transmission rates among arboviruses such as in chikungunya and West Nile virus [29].

Whole-genome sequencing (WGS) provides the ability to assess the intra-sample variation of a target genome within complex environmental samples, such as whole mosquito traps. The RAMPART workflow by the ARTIC network [30] is a WGS-based pipeline that has been used in molecular epidemiology studies of the Ebola virus outbreak of 2014–2016 in West Africa [31] and subsequently has been used for the Zika virus pandemic in South America [30], poliovirus [32] and SARS-CoV-2 [33]. The ARTIC network uses a targeted approach that utilises tiled PCR amplification and RAMPART for real-time reference mapping to identify the virus present in the sample [30, 34]. RAMPART is an end-to-end analysis system which incorporates commonly used programmes (minimap2 [35] and Porechop [36]) into a user-friendly GUI, allowing the user to monitor Oxford Nanopore MinION sequencing runs in real time. The annotated reads from RAMPART can then be processed downstream. Currently the post analysis for RAMPART can be run using InterARTIC GUI [37]. This pipeline combines BCFtools [38], medaka [39], nanopolish [40] and minimap2 [35] among other programmes to generate SNP called viral genomes from Nanopore data.

In this study we have customised InterARTIC and RAMPART workflows for RRV and applied them to field-collected mosquitoes from arbovirus surveillance traps that were homogenised for virus detection. In addition, we identified SNPs within a significant variable region of RRV and assigned viral haplotypes to inform the viral ecology in the Gippsland Lakes region of Victoria.

## Methods

### Ross River virus-positive material

**Fig. 1:**
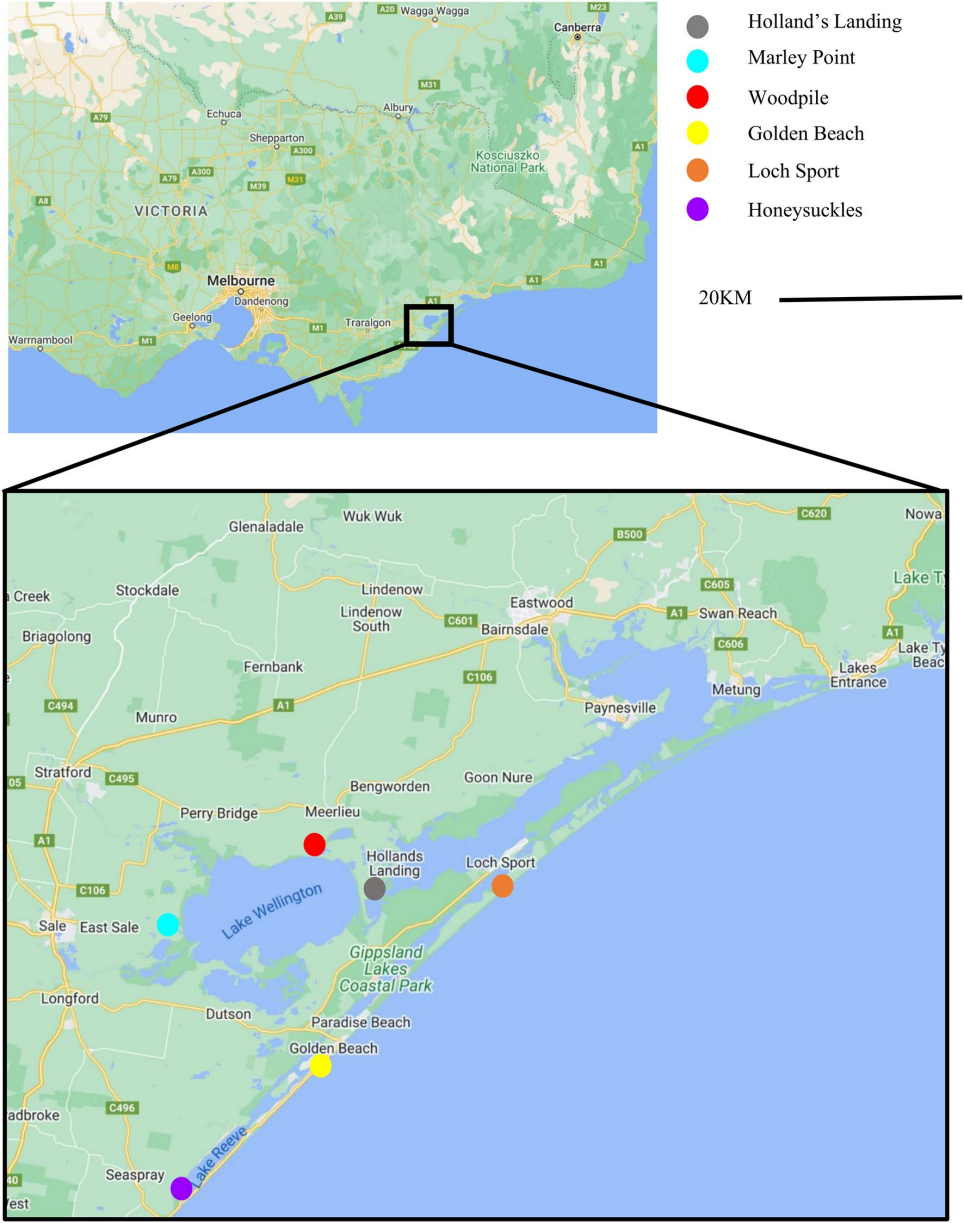
Map of agricultural Victoria mosquito sampling sites in Gippsland, Victoria. Map illustrates sampling sites from 2019 to 2022 which were used in this study. Maps derived from Google Maps website

RRV-positive mosquito whole trap grind homogenates and RRV cell culture-derived isolates used in this study are listed in Table 1; mosquito speciation breakdown for traps where available is provided (see Additional file 1). Mosquito whole trap grinds were prepared from overnight mosquito collections from sampling sites in Fig. 1, using previously described methods [41] and cell culture-derived isolates [42].

**Table 1:** List of positive mosquito traps used for analysis

Accession number	Sample name	Trap name	Number of mosquitoes	Year sampled	Location (see Fig. 1)
N/A	MP-20-Apr	20-01482-0019	1801	2020	Marlay Point
OQ355660	HL-20-Mar	20-01085-0007	282	2020	Hollands Landing
OQ355665	MP-20-Mar-1	20-01085-0027	1113	2020	Marlay Point
OQ355664	MP-20-Mar-2	20-01085-0028	1113	2020	Marlay Point
OQ355668	MP-20-Mar-3	20-01287-0004	4219	2020	Marlay Point
OQ355662	WP-20-Mar-1	20-01287-0008	1802	2020	Woodpile
OQ355663	WP-20-Mar-2	20-01287-0009	1802	2020	Woodpile
N/A	MP-20-Mar-4	20-01395-0006	2028	2020	Marlay Point
OQ355656	MP-20-Mar-5	20-01395-0007	2028	2020	Marlay Point
OQ355667	WP-20-Apr	20-01482-0018	2263	2020	Woodpile
		ARBO012-G4C (Batch1)	N/A		N/A
OQ355659	GB-20-Nov	20-05168-0033	4848	2020	Golden Beach
N/A	GB-20-Apr	20-01482-0023	N/A	2020	Golden Beach
OQ355661	GB-20-Apr	20-01544-0004	516	2020	Golden Beach
OQ355666	HS-20-Apr	20-01544-0003	252	2020	Honeysuckles
N/A	LS-22-Dec	22-00052-0034	6942	2022	Loch Sport
OQ355657	GB-21-Jan	21-00309-0024	2194	2021	Golden Beach
N/A	GB-20-Dec	20-05244-0010	N/A	2020	Golden Beach
OQ355658	HS-20-Dec	20-05244-0009	3368	2020	Honeysuckles
OQ355655	LS-21-Jan	21-00309-0026	4536	2021	Loch Sport
OQ355654	HS-22-Jan	22-00127-0032	1164	2022	Honeysuckles
		ARBO012-G4C (Batch2)	N/A		N/A

Positive Ross River virus homogenate traps used in this study, including the positive control (ARBO012), are listed twice as they were used in two separate sequencing runs. Positive homogenates are listed as full genome accession number, sample name used in this study, trap name used for the surveillance work and geographic location where the trap was collected

### RT-qPCR for Ross River virus

To confirm the presence of RRV and assess the relative amount of RRV genomic nucleic acid in a mosquito homogenate trap sample (using CT value), a RRV RT-qPCR-specific assay was applied to extracted RNA samples. This assay targets the E2 gene and produces an amplicon of 67 bp. RT-qPCR was performed on the extracted RNA samples as reported [42–44].

### Primer design for Ross River virus whole genome sequence tiling amplicon scheme

Primers were designed for the tiling amplicon scheme using the online portal PrimalScheme [30]. Eleven whole genome sequences were selected and uploaded as reference to PrimalScheme (Table 2); there were representatives of the four different RRV genotypes, with genotypes including G4A and G4B, which were recently detected in Victoria, as well as the historical genotypes (G2 and G1).

**Table 2:** Ross River virus isolates used for primer design*Homo sapiens*

Genbank accession	Virus name	Year	Genotype
GQ433359.1	T48	2009	G1
MK028845.2	Ross River virus/H.sapiens-wt/Australia/1972/14389	1972	G1
MK028844.2	Ross River virus/H.sapiens-wt/Australia/1994/ORegan	1994	G4B
MK028847.1	Ross River virus/A.vigilax-tc/Australia/1959/T48	1959	G1
MK028846.1	Ross River virus/A.camptorynchus-tc/Australia/1995/DC5692	1995	G2B
MK028843.1	Ross River virus/H.sapiens-wt/Australia/2009/PW14	2009	G4A
MW489504	Ross River virus isolate ARBO012	2013	G4B
MW517834	Ross River virus isolate ARBO231	2017	G4A
MW517836	Ross River virus isolate ARBO235	2017	G4A
MW489505	Ross River virus isolate ARBO113	2016	G4A
MW517835	Ross River virus isolate ARBO232	2017	G4A

The Ross River virus genomes and associated Genbank accession numbers and genotypes used to generate pan-genotype family primers using the PrimalScheme software

The ARBO012 (G4B, MW489504) sequence was set as the reference for PrimalScheme to represent a contemporary RRV sequence from the Wellington Shire, Victoria, Australia. The amplicon length was set at 1500 bp with neither the “High GC” nor “Pinned” options selected. The primer sequence output from PrimalScheme was then synthesised by IDT (Integrated DNA Technologies, IA, USA) as oligonucleotides with a total of nine primer pairs each producing 1500-bp-long overlapping amplicons (Table 3). The variable E2/E3 region of RRV was captured within a single amplicon (primer pair 7) for subsequent SNP and intra-trap viral diversity analysis. Primers were resuspended to a concentration of 100 µM with two primer pools, “even” and “odd”, prepared for the tiling application, following the methods of the ARTIC network [45]. In each of the two primer pools, the individual primer concentration was 0.015 µM per primer, with a final total pooled concentration of 100 µM. This was then diluted to make a working solution of 10 µM and used in the following PCR amplification reactions.

**Table 3:** Primers used for generating 1500-bp amplicons from RRV

Primer name	Primer sequence (5′–3′)	Location in RRV genome
RRV_New_1500_1_LEFT	ATGACCATGCTAATGCCAGAGC	
RRV_New_1500_1_RIGHT	ATTCCTGGGTGTCTCCACTACC	
RRV_New_1500_2_LEFT	CGTACTCTGGAGACCGAAACGA	
RRV_New_1500_2_RIGHT	ATGTTGTCATGCTCCTCTTGCC	
RRV_New_1500_3_LEFT	AGGCAGAAAGTGAATGAAAACCC	
RRV_New_1500_3_RIGHT	GACAACAGAGGGATGGCTACAC	
RRV_New_1500_4_LEFT	ATGAATGTCATCCACGCGGTAG	
RRV_New_1500_4_RIGHT	ATCAGACGAGAAGATGTACGCC	
RRV_New_1500_5_LEFT	GCACCTGAAGATCTGGAGGTAC	
RRV_New_1500_5_RIGHT	CAGTACACGGCATGCTATGACA	
RRV_New_1500_6_LEFT	CTCGGGGTTGACCAAGAACTAC	
RRV_New_1500_6_RIGHT	ACCCGAGTGACCATGTCTTTTG	
RRV_New_1500_7_LEFT	GAAGGTTTACCATCCCCACAGG	E2/E3
RRV_New_1500_7_RIGHT	AGAGTTAGGAGGGCCATCAGAC	E2/E3
RRV_New_1500_8_LEFT	CCAGTGACGGAAGAAGGGATTG	
RRV_New_1500_8_RIGHT	TTGGAATGTGAGTGGACAGCG	
RRV_New_1500_9_LEFT	TCTGTGGGACGAGAACAAAACC	
RRV_New_1500_9_RIGHT	ACTAAAGCTTACCGACGCATTGT	

Tiled PCR primer sequences for generating 1500-bp amplicons for whole genome amplification of Ross River virus

### Viral RNA extraction and reverse transcription for whole genome sequence tiling amplicon scheme

Viral RNA was extracted from mosquito whole trap grind homogenates (Table 1) and from cell culture-derived RRV material used as the positive control [42]. Fifty microlitres of RRV infected cell culture and mosquito whole trap grind homogenates was processed using a standard MagMax™ Viral RNA isolation preparation kit on the MagMax™ (Thermo Fisher Scientific, MA, USA) 24 Express processor. For every 50 µl of trap grind homogenate or virus cell culture, 65 µl of lysis buffer was mixed with 1 µl RNA carrier, 65 µl 100% isopropanol, 10 µl RNA beads and 10 µl RNA enhancer, provided in the MagMax™ kit. Two rounds each of washes one and two were used (150 µl each). The final extraction was eluted into 50 µl elution buffer.

Synthesis of cDNA was performed on the extracted RNA using 2 µl of 5X LunaScript RT SuperMix (New England Biolabs, MA, USA), which contains random hexamers, and this was combined with 8 µl of the extracted RNA and incubated at 25 °C for 2 min, 55 °C for 10 min followed by a final incubation at 95 °C for 1 min. The cDNA was kept at 4 °C until used for targeted enrichment of RRV using the tiled whole genome amplification scheme.

### PCR amplification for whole genome sequence tiling amplicon scheme

cDNA derived from the mosquito whole trap grind homogenates was PCR amplified based on the Midnight 1200 kb amplification method [46] with major modifications. For each sample, two reactions were prepared (“odd” and “even”, Table 3). Each reaction contained 2.5 µl of template cDNA, 9.6 µl of nuclease free water, 0.40 µl of one of the 100 µM primer pools and 12.5 µl Q5 Hot Start Hi-Fi 2X master-mix (New England BioLabs, MA, USA) and was PCR amplified under the following conditions: 98 °C for 30 s and 40 cycles of 98 °C for 15 s with 65 °C for 7 min to enrich for RRV.

### Library preparation and nanopore sequencing of Ross River virus

The amplicons were sequenced using a combination of the GunIt Method [45] and LoCost Method [47] with modifications. The 24 individual PCR reactions (two PCR tiled reactions per mosquito homogenate sample) were combined and diluted into 12 50-µl pools containing 2.5 µl of each PCR tiled reaction per sample and 45 µl nuclease free water. For each of the 12 pooled PCR reactions, 7.5 µl of the corresponding diluted PCR product, 5 µl of nuclease free water, 1.75 µl of Ultra End II Prep Reaction Buffer (New England BioLabs, MA, USA) and 0.75 µl Ultra End II Prep Enzyme Mix (New England BioLabs) were combined and incubated at room temperature for 15 min, 65 °C for 15 min and incubated on ice for 1 min.

To generate barcoded samples for sequencing, 4.2 µl of the PCR tiled template was combined with 3 µl water, 2.5 µl of the NB barcode (SQK-LSK109 and EXP-NBD104, Oxford Nanopore Technologies, UK), 10 µl Blunt/TA Ligase Master Mix (New England BioLabs) and 3 µl water. The mix was incubated at room temperature for 20 min, 65 °C for 10 min and on ice for 1 min.

Twenty microlitres of the barcoded reactions was pooled to form the final library for sequencing and combined with ProNex beads (Promega, WI, USA) at 0.7× the amount of pooled volume (168 µl of beads). The mixture was incubated for 5 min at room temperature and the supernatant removed when clear. The beads were washed twice by resuspending in 250 µl Short Fragment Buffer (Oxford Nanopore Technologies, UK), mixed and pelleted and the supernatant discarded. The beads were then washed in 200 µl 70% ethanol (room temperature), being careful not to disturb the pellet. The ethanol was removed and pellet dried for approximately 1 min or until shiny. The pellet was resuspended in 31 µl Elution Buffer (Oxford Nanopore Technologies), incubated for 2 min and transferred into a clean Eppendorf tube, forming the nucleic acid library for sequencing. A 1 µl aliquot of the library was quantified on Qubit Flex (Thermo Fisher Scientific, MA, USA) using a dsDNA High Sensitivity Kit.

For ligation of sequencing adaptors to the barcoded samples the total of the end-repaired MinION library (30 µl) was combined with 10 µl of the NEBNext Quick Ligation Reaction Buffer (5×), 5 µl of Adapter Mix (AMII) and 5 µl Quick T4 DNA Ligase and incubated at room temperature for 20 min. ProNex beads, at a ratio of 0.7× the library volume, were added to the above mix (35 µl of beads) and incubated for 5 min at room temperature with the supernatant removed when clear. The pelleted beads were washed with 250 µl Short Fragment Buffer (Oxford Nanopore Technologies, UK); the supernatant was removed and washed again. The pellet was resuspended in 13 µl elution buffer (Oxford Nanopore Technologies), incubated for 2 min at room temperature and the eluate collected for sequencing. One microlitre of the final sequence adaptor ligated library was quantified on a Qubit using the dsDNA kit.

The eluted sample was then loaded onto a pre-prepared MinION flow cell, following standard MinION loading and sequencing procedures (Genomic DNA by Ligation, version GDE_9063_v109_revAJ_14Aug2019).

### Bioinformatic analysis of Ross River virus whole genome nanopore data

Both RAMPART [34] and InterARTIC [37] were modified for use with RRV following the instructions on the respective GitHub pages. Primer files were generated for this study (1500-bp amplicon set) as well as the RRV reference sequence that was annotated and loaded in the primer.json file for RAMPART to utilise. An array of RRV genomes covering all genotypes, including extant viruses (G1 and G2), was also loaded into the references.fasta file for RAMPART to perform reference mapping steps.

RAMPART was used to monitor the progression of the MinION sequencing, initially to identify any mixed genotypic samples and to genotype the samples by reference mapping back to the references.fasta file.

### Generation of Ross River virus reference genome

To compare data across whole mosquito trap samples, all generated consensus sequences needed to be normalised. To reduce any potential bias towards genotypes in the reference mapping stage, a generic consensus RRV sequence was created. The consensus was generated using 123 partial and full genomes downloaded from NCBI (see Additional file 2). All sequences were loaded into Geneious (V11.0.11) and aligned using the MUSCLE algorithm. Sequences were then trimmed to the shortest genome from both the 5′ and 3′ ends. The consensus of the trimmed genome sequences (11 296 nt in length) was exported and used as a reference for all following analyses (henceforth referred to as “RRV reference sequence”). The E2/E3 region of the RRV genome used for fine-scale analysis corresponded to nucleotides 8222–9743 of this reference sequence.

### Generation of consensus whole genome sequences of Ross River virus from mosquito whole trap homogenate grinds

The InterARTIC nanopolish pipeline was run on the mosquito whole trap homogenate samples (post-24 h of MinION sequencing) using the parameters defined by the “Custom Virus” option. The resulting BAM file was used for downstream processing. Only mosquito homogenate trap samples that generated all nine amplicons were used for whole genome sequence analysis. Mosquito homogenate trap samples that generated the seventh amplicon that corresponds to the E2/E3 region of the RRV genome were used for minor SNP analysis and haplotype analysis.

The BAM file was run through the following BCFtools [38] (V 1.14-GCC-11.2.0) command for whole genome generation [bcftools mpileup -a INFO/AD -O u -d 10,000 -L 9000 -f reference.fasta sorted/bam/from/nanopolish.sorted.bam| bcftools call -mv -O u | bcftools norm -O u -f reference.fasta | bcftools filter -O u -i'%QUAL > 180' | bcftools view -O v -i "(INFO/AD[1]/INFO/DP) > 0.45" > barcode.vcf | bcftools consensus barcode.vcf > barcode_consensus.fasta]. The resultant whole genome consensus sequence of each individual mosquito homogenate trap sample was used in phylogenetic analysis.

### Phylogenetic analysis of whole genome and haplotypes

A maximum likelihood (ML) phylogenetic analysis was performed using (i) whole genome consensus sequences gathered from NCBI, (ii) sequences generated from the 16 RRV-positive whole trap mosquito trap grinds, (iii) two positive controls (ARBO012-MinION01 and ARBO012-MinION02; Table 2) and (iv) sequences included in a previous RRV study [19]. The ML tree was generated by MEGA11 [48], after alignment with MAFFT [49], using the General Time Reversable module in MEGA11 and a bootstrap value of 1000.

### Minor SNP analysis within E2/E3 region of the Ross River virus genome

Sequence analysis of the E2/E3 region of the RRV genome was performed with both SAMtools [38] (1.15-GCC-11.2.0) and iVar [50] with the following command [samtools mpileup -aa -A -d 1,000,000 -B -Q 0 -f reference.fasta bam/file/from/nanopolish/.sorted.bam -r NCBI:8222-9743 | ivar variants -p sample_name -q 20 -t 0.03 -r reference.fasta -m 30]. Subsequent .tsv files were examined and any indels were removed as these were deemed unreliable for fine-scale analysis. The remaining SNPs were used for fine-scale analysis and were compared across traps to examine consistency and potential variation. SNPs were transferred to a .csv file where they were then used for manual identification of haplotypes across the traps examining frequencies and nucleotide positions. SNPs were called using a lower threshold than the whole genome analysis (3% of reads vs. standard setting for BCFtools).

### Haplotype analysis using the E2/E3 region

Haplotyping of the trap samples was done through visual inspection of the alignment paying particular attention to minor SNP frequencies identified via the .csv file. Specific haplotypes for each trap were determined by the presence or absence of certain SNPs in the E2/E3 region of the RRV genome until all SNPS from all isolates were attributed to haplotypes. Reads were manually inspected to ensure the appropriately assigned haplotype SNPs were present using IGV (Version 2.5.0 [51–53]) and Tablet (Version 1.21.02.08 [54]). Phylogenetic analysis of haplotypes was performed in MEGA11 as outlined above in the WGS analysis.

## Results

### Rapid, accurate generation of Ross River virus whole genome sequences from field whole mosquito trap homogenates

RAMPART and InterARTIC analyses indicated that the RRV, present in all 20 mosquito field trap homogenates, collected from the Wellington Shire, Victoria, belonged to a single genotype, G4A. Sixteen of the mosquito field trap homogenates produced sufficient coverage across all nine amplicons (indicated by the presence of all nine amplicons at a coverage > 20×) to enable the assembly of a whole genome sequences of RRV and subsequent phylogenetic analysis.

**Fig. 2:**
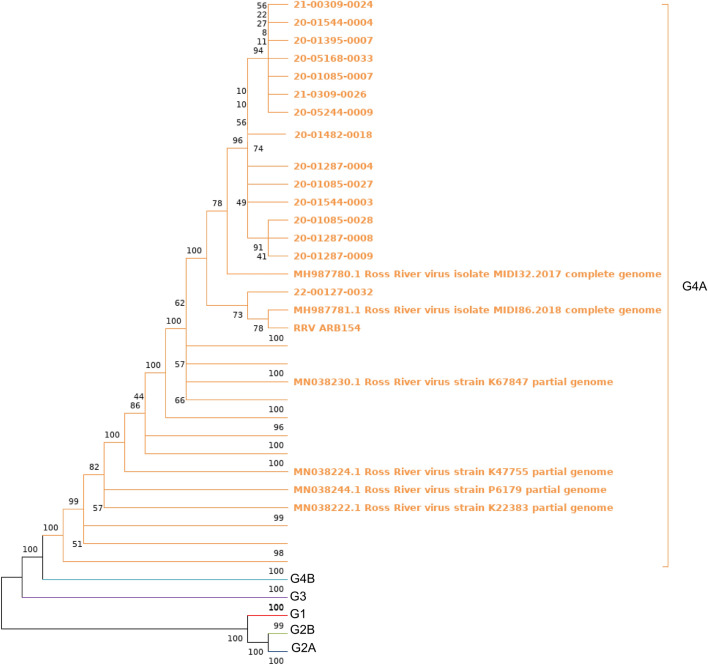
Maximum likelihood (ML) phylogenetic tree. Phylogenetic tree of whole RRV genomes using a GTR model, bootstrap 1000. The tree uses genome consensus nucleotide sequences from 16 mosquito traps collected from six locations. Included in the analysis is the RRV-positive sequencing control (a cell culture-derived isolate, ARBO012) that was included in both MinION sequencing runs. Sequences are distinguished in their genotypes by colour

Maximum likelihood phylogenetic analysis of the 16 whole genome consensus sequences confirmed clustering of the samples within G4A (Fig. 2). There was no spatial-temporal clustering of the 16 genomic consensus sequences analysed from Wellington Shire between 2020 to 2022 (Fig. 2).

There were no identical whole genome trap sequences of RRV between the mosquito homogenate traps. The most diverse RRV whole genome sequences were between the trap HS-22-Jan and MP-20-Mar-5, which differed by 34 nucleotides across the whole genome (0.3% divergence across 11 296 nt). The most similar RRV whole genome sequences were from traps MP-20-Mar-2 and WP-20-Mar-1, which differed by only one nucleotide over the whole genome (Fig. 2).

### SNP analysis and detection of Ross River virus haplotypes

Of the 20 traps sequenced in this study, 18 produced a full length E2/E3 amplicon that was used for the detection of SNPs at a frequency of > 3% of all reads to support fine-scale haplotype analysis.

The positive control (ARBO012) sequence data generated from two sequencing runs were both identical to the previously generated (Illumina-based) reference sequence across the E2/E3 amplicon, indicating that there was no inter-run variation between SNP analysis in the variable region.

**Fig. 3:**
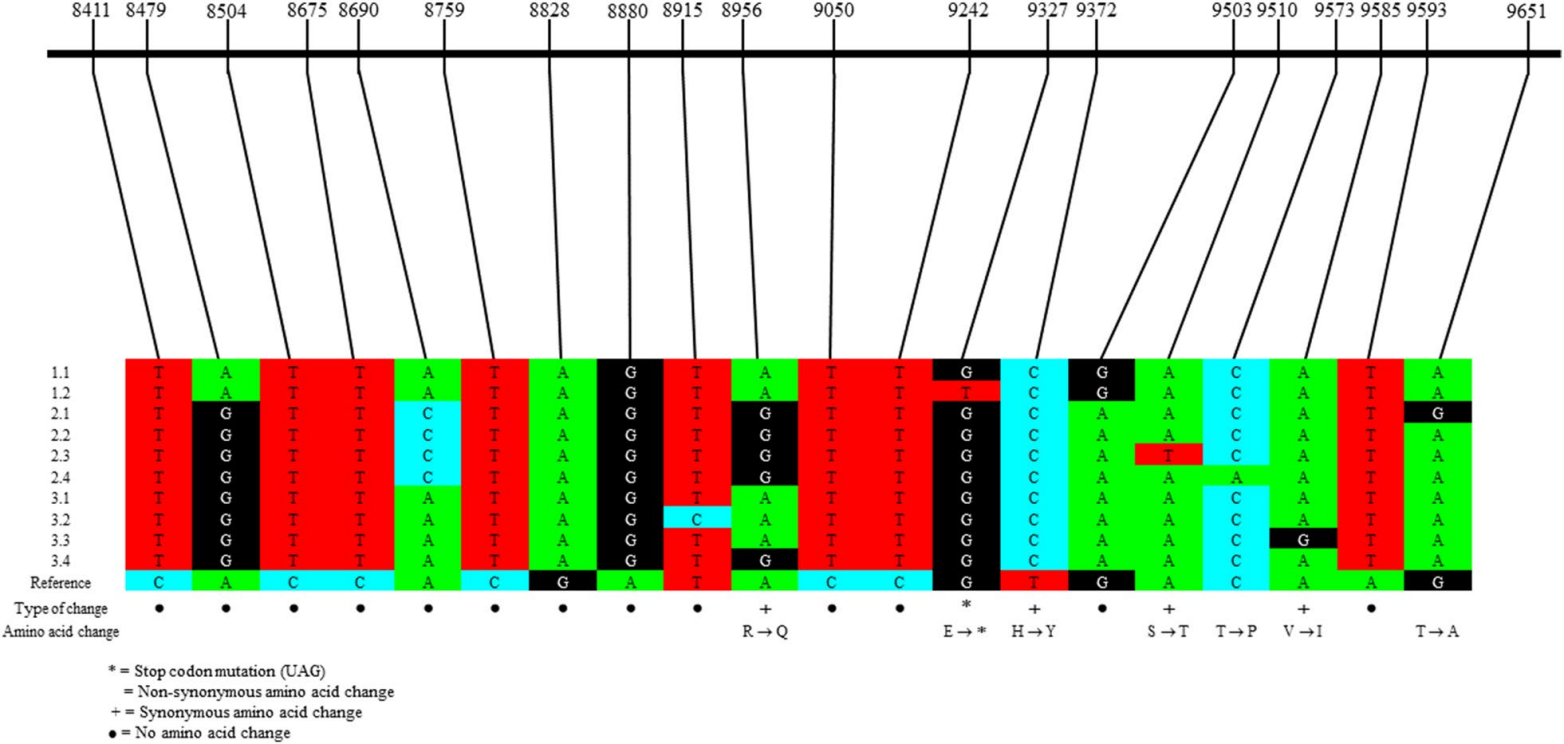
Intra-mosquito trap RRV E2/E3 diversity and genetic analysis of different haplotypes observed across Wellington Shire. Schematic illustration of Amplicon 7 (E2/E3 region 8222–9743nt) with all haplotype mutations denoted from the generic consensus RRV sequence. Haplotypes (1.1 to 3.4) are listed on the left. MEGAX was used to visualise the alignment and translate the nucleotide bases into amino acid residues

**Fig. 4:**
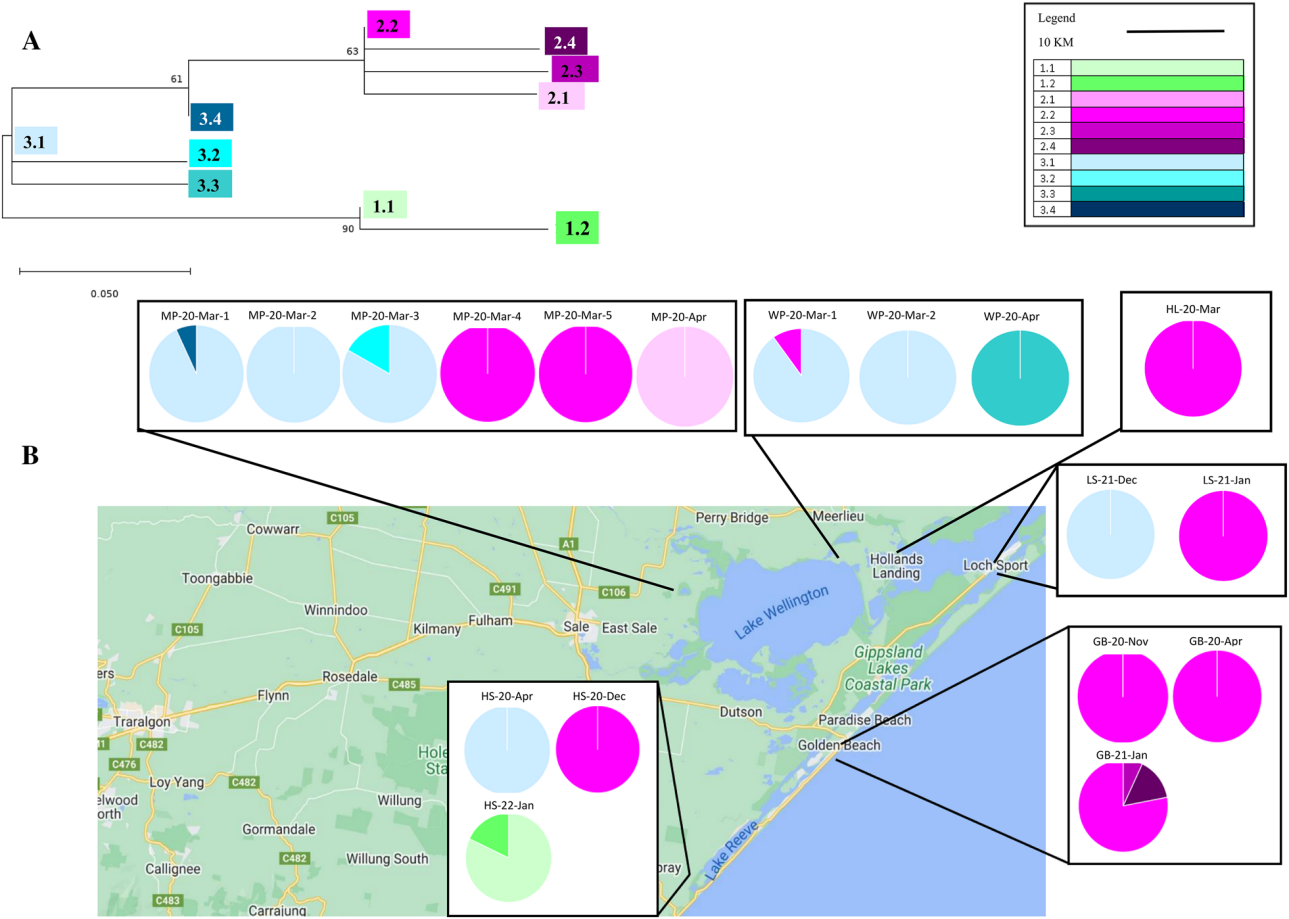
Temporal intra-mosquito trap diversity from RRV E2/E3. **A** Maximum likelihood (ML) phylogenetic tree, GTR model, bootstrap 1000 of the RRV haplotypes generated using MEGAX. Haplotypes are distinguished in their genotypes by colour. All are haplotypes of the same genotype, G4A. **B** Graphical illustration of spatial-temporal RRV E2/E3 haplotype variation. Each trap is represented by a pie chart with the proportion and number of haplotypes illustrated with corresponding colours. Maps derived from Google Maps website

Twenty SNPs were detected across the 1500-bp E2/E3 amplicon from 18 traps. Seven of the 20 SNPs resulted in amino acid changes, with one of the seven SNPs generating a stop codon (Fig. 3). From the 20 SNPs, ten unique haplotypes were determined (Fig. 3). Phylogenetically, the ten different haplotypes were represented in three different clades (labelled 1–3). RRV haplotypes lacked any spatial-temporal structure, with the most prominent haplotype, 2.2, detected in nine separate traps, across the three years sampled and in all six locations (Fig. 4B, Table 4). This haplotype was detected at GB three times over a 10-month period (between April 2020 and January 2021; Table 4). The second most common haplotype was 3.1, detected in seven traps across 1 year in the locations MP, WP and LS (Fig. 4B, Table 4). This haplotype was detected at MP twice over 13 days in March 2020 in two different trapping events. Eight haplotypes were only seen in one trapping event once (1.1 and 1.2 in HS, 3.2, 3.4 and 2.1 in MP, 3.3 in WP, and 2.3 and 2.4 in GB; Fig. 4B, Table 4).

**Table 4:** Detected haplotypes in the Wellington Shire region across

		Location					
		GB	HS	LS	MP	WP	HL
Haplotypes	1.1		10/01/2022				
	1.2		10/01/2022				
	2.1				3/4/2020		
	2.2	9/4/2020–25/01/2021	2/12/2020	25/01/2021	27/03/2020	19/03/2020	6/3/2020
	2.3	25/01/2021					
	2.4	25/01/2021					
	3.1		9/4/2020	28/12/2021	6/03/2020–19/03/2020	19/03/2020	
	3.2				19/03/2020		
	3.3					3/4/2020	
	3.4				6/3/2020		

Detected haplotypes of Ross River virus from the Gippsland Lake region, Victoria, with time frames of arbovirus surveillance programme screening dates included for added epidemiological information. Haplotype is listed to the left with any detection denoted by the time that the trap was screened

### Intra-mosquito trap diversity of E2/E3 region of Ross River virus

Mixed haplotypes were detected in five of the 18 traps. In each of the five mixed haplotype traps, frequency percentage of minor haplotypes varied from 0.7 to 18%. Within four mixed traps only two haplotypes were seen, with one trap from GB in 2022 showing a mixture of three haplotypes. The SNP that resulted in the stop codon was present in haplotype 1.2 and was only found once within a trap that had two haplotypes and was detected at a frequency of 17.6% of the E2/E3 reads in that trap.

## Discussion

Whole genome sequencing and genomic epidemiology are increasingly being used to understand viral diversity during an epidemic or outbreak. With the ability to detect minor variants and track these variants across time and between locations, genomic data have proving useful to monitor epidemics. The analysis of viral genome sequences can inform an understanding of the ecology and transmission of viruses [55]. Additionally, analysis of genome sequences can identify potential amino acid or nucleotide changes that may affect virulence and subsequently the suitability of diagnostic assays and vaccines [56]. In this study, we have developed a novel bioinformatic pipeline for RRV, an important mosquito-transmitted arbovirus in Australia. This pipeline was then used to analyse a collection of RRV-positive whole mosquito trap homogenates to understand the spatial-temporal genetic structure of the virus within the Wellington Shire, Gippsland, Victoria, Australia. The MinION sequencing platform from Oxford Nanopore Technologies platform was selected for this study because of its ability to generate longer sequence reads from viral genomes that enable fine-scale analysis and the resolution of viral haplotypes within complex environmental samples [50]. This contrasts with short-read sequencing platforms such as Illumina, as sequence diversity is limited to the read length, hindering the ability to confidently assess single individual genomes and viral haplotypes [57].

G4 is the most common contemporary genotype of RRV in Australia [17, 19, 58]. G4A and G4B have both been detected in Victoria, Queensland and Western Australia [19, 59]. From the five Victorian whole genome sequences analysed previously, spatial clustering had been detected, with G4B detected only in the Gippsland Lakes area and G4A detected in inland Victoria [59]. In this study using RAMPART, amplicon tiling amplification and the established viral processing pipeline from the ARTIC group [34], the analysis of genomic sequences from an additional 20 whole mosquito trap grinds revealed that G4A is found in Gippsland Lakes. This is an interesting observation as, until this study, only G4B had been seen in Gippsland, in contrast to Western Australia, where both G4A and G4B have been detected spanning north, south and central regions for many years [19]. The apparent appearance of G4A in Gippsland in this study may reflect previous under-sampling and generation of WGS for subsequent analysis.

The lack of spatial-temporal viral structure of RRV in the Gippsland Lakes area and the detection of ten distinct viral haplotypes are consistent with heterogeneous viral populations in this area. The detection of a heterogenous viral population and lack of spatial-temporal structure in the Gippsland Lakes region are not surprising given that many different vertebrate hosts and vector species can be involved in RRV transmission [6, 7, 60] and that they are present in the local Wellington Shire [61]. In addition, the expansive saltmarsh wetlands facilitate productive breeding sites for the salt marsh mosquitoes *Ae. camptorhynchus* and *Ae. vigilax*, with reported flight ranges of 3 [62]—6 km [63] and up to 9 km [64] (excluding wind-assisted dispersal).

Transovarial transmission of arboviruses (viral transfer via mosquito eggs) can often be observed in field-caught male mosquitos that would not have consumed a blood meal; hence, the only method for these mosquitoes to attain the arbovirus is directly from the mother [9]. This has previously been detected for other arboviruses [9] (including Japanese encephalitis virus [65] and Eastern equine encephalitis virus [66]) and specifically seen in *Ae. vigilax* for RRV [67]. The repeated detection of haplotype 2.2 in GB over a span of 9 months (April 2020–January 2021) suggests that RRV overwintered in the eggs of mosquitoes [68], as this is below the reported nucleotide substation rate for RRV [19].

The SNP 9327 that produced an amino acid change in haplotype 1.2 resulting in a stop codon was detected at a low frequency of 17.6% in a whole trap grind sample. The appearance of the stop codon (SNP 9327) in a mixed haplotype sample was unexpected. However, reports of truncated proteins and stop codons in structural proteins have been previously described [69, 70]. Specifically, a frameshift mutation in the S1 gene of SARS-CoV-2 spike protein resulted in a truncated S1 protein at a low percentage. As there was a large percentage of fully formed S proteins, it was hypothesised that functional and available S1 protein would be acquired from the functioning viruses in the viral quasispecies swarm [69]. A similar stop codon has been detected in the nsP3 protein of O’nyong’nyong virus, an alphavirus similar to RRV, and it has been hypothesised that the presence of the stop codon has altered the infectivity of the virus in the *Anopheles gamdiae* host vector [70].

When using RAMPART and InterARTIC, only the genotype and the whole genome sequence will be identified. To facilitate viral haplotype analysis, fine-scale minor SNPs representing quasispecies above a threshold of 3% of reads having an alternative nucleotide to the reference had to be used. In this study, we developed a novel bioinformatic pipeline using iVar [50] and visual assessment of individual linked SNPs over the E2/E3 region to measure intra-host variation.

SNP analysis programmes such as BCFtools call [38], LoFreq [71], FreeBayers [72], WhatsHap [73, 74], VirStrain, HaploFlow, HaploClique, Shorah, nanopolish [75], etc., were deemed not suitable for the analysis as they apply a threshold level that is too high to detect minor alleles and instead report a consensus from the trap rather than the representative genome sequences within the viral swarm.

The full genome primers developed in this assay covered only the CDS of RRV. The exclusion of the 3′ and 5′ UTRs may result in missed mutations in the virus, and this is a limitation of the study.

## Conclusions

With the ability to detect minor alleles from populations of mosquitoes, a more comprehensive picture of circulating RRV strains can be understood. Increased surveillance could show the spread of certain viral haplotypes and could be applied to understand population size within a given season. The methods developed here could also be applied to other mosquito-borne arboviruses of public health significance.

## Supplementary Information

**Additional file 1.** Speciation of mosquitoes from sample traps. Tabulated data of all mosquito species and numbers detected in traps used for RRV sequencing. Only traps that had applicable data are shown.

**Additional file 2.** RRV genomes used for generation of one full genome for standardisation; 123 RRV whole genome sequences used to generate a singular consensus file to standardised all generated RRV whole genome sequences against. All sequences are listed with their accession number and were downloaded from NCBI.

## Data Availability

The datasets generated from this study are available on request from the corresponding author.

## Abbreviations

Ae: Aedes
cDNA: Complementary deoxyribonucleic acid
CDS: Coding sequence
Ct: Cycle threshold
Cx: Culex
dsDNA: Double-stranded deoxyribonucleic acid
E2/E3: Envelope protein 2/3
GB: Golden Beach, Wellington, Victoria
GUI: Graphical User Interface
HL: Holland's Landing, Wellington, Victoria
HS: Honeysuckles, Wellington, Victoria
LS: Loch Sport, Wellington, Victoria
MP: Marley Point, Wellington, Victoria
PCR: Polymerase chain reaction
RAMPART: Read Assignment, Mapping, and Phylogenetic Analysis in Real Time
RNA: Ribonucleic acid
RRV: Ross River virus
RTqPCR: Reverse transcription quantitative polymerase chain reaction
SNP: Single nucleotide polymorphism
UTR: Untranslated region
WGS: Whole genome sequencing
WP: Woodpile, Wellington, Victoria
